# The impact of trans-catheter aortic valve replacement induced left-bundle branch block on cardiac reverse remodeling

**DOI:** 10.1186/s12968-017-0335-9

**Published:** 2017-02-22

**Authors:** Laura E. Dobson, Tarique A. Musa, Akhlaque Uddin, Timothy A. Fairbairn, Owen J. Bebb, Peter P. Swoboda, Philip Haaf, James Foley, Pankaj Garg, Graham J. Fent, Christopher J. Malkin, Daniel J. Blackman, Sven Plein, John P. Greenwood

**Affiliations:** 1 Multidisciplinary Cardiovascular Research Centre (MCRC) & Leeds Institute of Cardiovascular and Metabolic Medicine (LICAMM), University of Leeds, Clarendon Way, Leeds, LS2 9JT UK; 2 Department of Cardiology, Leeds Teaching Hospitals NHS Trust, Leeds, LS1 3EX UK

**Keywords:** Aortic valve stenosis, Trans-catheter aortic valve implantation, Left bundle branch block, Ventricular ejection fraction, Ventricular remodeling, Cardiovascular magnetic resonance

## Abstract

**Background:**

Left bundle branch block (LBBB) is common following trans-catheter aortic valve replacement (TAVR) and has been linked to increased mortality, although whether this is related to less favourable cardiac reverse remodeling is unclear. The aim of the study was to investigate the impact of TAVR induced LBBB on cardiac reverse remodeling.

**Methods:**

48 patients undergoing TAVR for severe aortic stenosis were evaluated. 24 patients with new LBBB (LBBB-T) following TAVR were matched with 24 patients with a narrow post-procedure QRS (nQRS). Patients underwent cardiovascular magnetic resonance (CMR) prior to and 6 m post-TAVR. Measured cardiac reverse remodeling parameters included left ventricular (LV) size, ejection fraction (LVEF) and global longitudinal strain (GLS). Inter- and intra-ventricular dyssynchrony were determined using time to peak radial strain derived from CMR Feature Tracking.

**Results:**

In the LBBB-T group there was an increase in QRS duration from 96 ± 14 to 151 ± 12 ms (*P* < 0.001) leading to inter- and intra-ventricular dyssynchrony (inter: LBBB-T 130 ± 73 vs nQRS 23 ± 86 ms, *p* < 0.001; intra: LBBB-T 118 ± 103 vs. nQRS 13 ± 106 ms, *p* = 0.001). Change in indexed LV end-systolic volume (LVESVi), LVEF and GLS was significantly different between the two groups (LVESVi: nQRS -7.9 ± 14.0 vs. LBBB-T -0.6 ± 10.2 ml/m^2^, *p* = 0.02, LVEF: nQRS +4.6 ± 7.8 vs LBBB-T -2.1 ± 6.9%, *p* = 0.002; GLS: nQRS -2.1 ± 3.6 vs. LBBB-T +0.2 ± 3.2%, *p* = 0.024). There was a significant correlation between change in QRS and change in LVEF (*r* = -0.434, *p* = 0.002) and between change in QRS and change in GLS (*r* = 0.462, *p* = 0.001). Post-procedure QRS duration was an independent predictor of change in LVEF and GLS at 6 months.

**Conclusion:**

TAVR-induced LBBB is associated with less favourable cardiac reverse remodeling at medium term follow up. In view of this, every effort should be made to prevent TAVR-induced LBBB, especially as TAVR is now being extended to a younger, lower risk population.

## Background

The aortic valve lies close to the electrical conduction system of the heart and is prone to damage at the time of aortic valve intervention, often manifesting as new left-bundle branch block (LBBB). New LBBB is infrequent following surgical aortic valve replacement [1], but much more common following trans-catheter aortic valve replacement (TAVR) with reported rates of up to 65%, depending on valve design [2]. TAVR-induced left-bundle branch block (LBBB-T) has been linked to reduced survival [3–5] and increased hospitalisation [6], in keeping with population based studies suggesting reduced overall survival in healthy individuals with LBBB [7] and in patients with heart failure and LBBB [8]. The mechanism for this increased mortality is debated; one hypothesis is that LBBB-T is a precursor to further more lethal conduction abnormalities [9], another is that LBBB-T leads to abnormal left ventricular (LV) remodeling and ultimately heart failure death via a LBBB-induced cardiomyopathy [10]. Current evidence on the impact of LBBB-T on cardiac reverse remodeling is limited to echocardiographic studies, with a heterogeneous patient mix including those with post-procedural permanent pacemaker implantation, trans-apical access route and unmatched patient groups [10–12], all of which are potential confounders in the reverse remodeling process. The impact of LBBB-T on cardiac reverse remodeling has never been investigated using cardiovascular magnetic resonance (CMR), which is the reference standard technique for LV mass and volume quantification, allowing important differences to be determined with a small sample size [13]. Furthermore, the novel technique of feature tracking allows accurate estimation of global longitudinal strain (GLS) and inter- and intraventricular dyssynchrony which are of interest in this population and may be able to assess the impact of LBBB on cardiac function beyond simple mechanical dyssynchrony [14].

We hypothesised that LBBB-T 1) negatively impacts on cardiac reverse remodeling at 6 m follow up and 2) is associated with inter- and intra-ventricular dyssynchrony compared with a matched ‘control’ population with a narrow QRS (nQRS) post-TAVR.

## Methods

### Patient selection

We evaluated 88 patients undergoing either Boston Lotus (Boston Scientific Corporation, Natick, MA) or Medtronic CoreValve (Medtronic Inc., Minneapolis, Minnesota) TAVR for severe symptomatic aortic stenosis at a single tertiary centre from April 2009 to April 2015. Exclusion criterion included pre-existing QRS prolongation (>120 ms) or contra-indication to CMR scanning. Decision for TAVR was taken by a multi-disciplinary heart team in accordance with international guidance [15]. Trans-femoral was the default approach with other techniques (subclavian and carotid) employed if femoral access was unsuitable.

### Matching

**Fig. 1:**
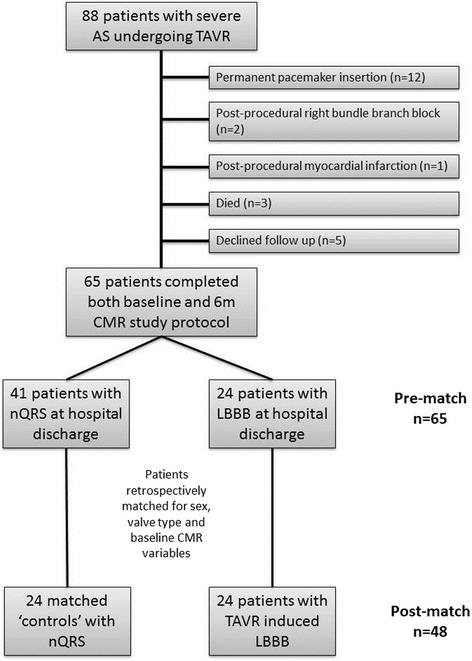
Patient recruitment and retrospective matching methodology. AS: Aortic stenosis. TAVR: Trans-catheter aortic valve replacement. CMR: Cardiovascular magnetic resonance. nQRS: Narrow QRS. LBBB: Left bundle branch block

24 patients with LBBB-T were identified. These were matched with 24 patients with a nQRS post-procedure for sex, valve type, and baseline CMR variables known to impact on reverse remodeling following TAVR including LV ejection fraction (LVEF), indexed LV mass and indexed LV end diastolic volume (LVEDVi) [16] (Fig. 1).

### Electrocardiographic data

12-lead electrocardiogram recordings acquired immediately prior to TAVR and at the time of post-procedure hospital discharge were reviewed by a single author blinded to clinical and procedural data. Heart rhythm, PR interval and QRS duration were recorded. LBBB-T was defined as post-procedural v1-negative QRS complex with a duration of >120 ms and a notched or slurred R wave in at least one of the lateral leads (I, aVL, V_5_, V_6_) [17].

### CMR protocol

Details of the CMR pulse sequence acquisition protocol have been published previously [16]. Briefly, identical CMR scans were obtained at baseline and 6 m following TAVR using a 1.5 T scanner (Intera, Philips Healthcare, Best, Netherlands or Avanto, Siemens Medical Systems, Erlangen, Germany). Multi-slice, multi-phase cine imaging was performed using a standard steady-state free precession pulse sequence in the short axis (10 mm thickness, 0 mm gap, 30 phases, 192 by 192 matrix, typical field of view 340 mm) to cover both ventricles. Standard 2, 3 and 4 chamber cine images were also acquired. Through-plane velocity encoded phase contrast imaging was performed perpendicular to the aortic valve jet at the aortic sinotubular junction (VENC 250–500 cm/s, retrospective gating, slice thickness 6 mm, 40 phases). Late gadolinium enhancement (LGE) imaging (10–12 short axis slices, 10 mm thickness, matrix 240x240, 320–460 mm field of view) was performed with inversion time individually adjusted according to TI scout, 10–15 min after 0.2 mmol/kg of gadoteric acid (Dotarem, Guerbet, Villepinte).

### CMR analysis

CMR analysis was performed by a single experienced operator blinded to clinical data using cmr^42^ (Circle Cardiovascular Imaging, Alberta, Canada). Endocardial and epicardial contours were manually contoured at end-diastole and end-systole with papillary muscles and trabeculations excluded to allow the calculation of ventricular volumes (summation of discs methodology) and mass (epicardial volume - endocardial volume multiplied by myocardial density (1.05 g/cm^3^)). Values were indexed to body surface area. Post-procedural myocardial infarct was determined by direct comparison of pre- and 6 m CMR LGE acquisitions. Fibrosis mass was quantified using a threshold of 5 standard deviations technique [18]. Left atrial volume was calculated using the biplane area-length method [19]. Aortic flow was quantified using cross-sectional phase contrast images with the slice positioned at least 10 mm above the aortic prosthesis and contouring of the aortic lumen to provide a regurgitant fraction (%). Longitudinal right ventricular function was measured at the lateral tricuspid annulus in the 4 chamber cine view as the distance between end systole and end diastole. Feature tracking analysis was performed on cine imaging of the mid ventricular short axis slice at the papillary muscle level to determine time to peak LV and right ventricular radial strain and the 4 chamber cine to measure global longitudinal strain. Interventricular dyssynchrony was the difference between time to peak radial strain of the right ventricular free wall and the lateral LV wall (an average of segments 11 and 12 of the American Heart Association 17 segment model). Intraventricular dyssynchrony was the difference between time to peak radial strain of the LV septal (an average of segments 8 and 9) and lateral LV segments (an average of segments 11 and 12). Segments with LGE indicating previous myocardial infarction were excluded from radial strain analysis. For the assessment of inter-observer variability, two independent investigators analysed LV volume, mass and function, GLS and time to peak radial strain on a random selection of 10 patients. For intra-observer variability a similar dataset from 10 patients was analysed twice by one investigator one month apart. The coefficient of variation was calculated by dividing the standard deviation of the differences between measurements by their mean and expressed as a percentage. Inter-observer variability was 1.4, 4.5, 3.7, 9.2 and 12.6% and intra-observer variability was 6.8, 2.6, 5.0, 2.6%, 6.8 and 9.1% for LVEDV, LV mass, LVEF, GLS and time to peak radial strain respectively.

### Statistical analysis

Statistical analysis was performed using SPSS version 22 (IBM, Armonk, New York). Categorical data were presented as numbers (percentages) and compared using the Pearson Chi squared test. Continuous variables were expressed as mean ± SD and were tested for normality using the Shapiro-Wilks test. Data were compared using Students *t* Test (for normally distributed data) and the Mann-Whitney or Wilcoxen signed rank test (for non-normally distributed data). Linear regression analysis (Enter model) was performed to establish univariate and multivariate predictors of change in LVEF and GLS post-procedure. Univariate predictors with *P* < 0.05 were included in the multivariate analysis. *P* < 0.05 was considered statistically significant. Using CMR, in order to detect a 3% difference in LVEF with a 90% power and an α error of 0.05 a sample size of at least 12 patients in each arm was required [13].

## Results

Eighty-eight patients were recruited. Patients undergoing post-procedure permanent pacemaker implantation (*n* = 12), those with post-procedure right bundle branch block (*n* = 2) and those with CMR LGE evidence of post-procedural myocardial infarction (*n* = 1) were excluded from analysis. In addition, 3 patients died within the 6 m follow up period and 5 patients declined follow up (Fig. 1). 24 patients with LBBB-T and 41 patients with nQRS on discharge electrocardiogram completed both baseline (median 1 day pre-procedure, IQR 1 day) and 6 m scans (median 181 days, IQR 20 days) and were available for retrospective matching in a 1:1 fashion (Fig. 1). 48 patients were included in the final analysis, 24 with LBBB-T and 24 with nQRS. Demographic, clinical, procedural and baseline CMR details for each group are shown in Table 1. 14 (29%) patients underwent Lotus valve and 34 (71%) patients underwent Medtronic CoreValve implantation. Balloon valvuloplasty was performed in 43 (90%) patients. Mean valve size was 28 ± 2 mm, procedure time 164 ± 52mins and contrast dose 153 ± 61 ml. Access approach was femoral in 43 (90%) patients, subclavian in 4 (8%) patients and carotid in one patient.

**Table 1:** Demographic, clinical and baseline CMR details of the nQRS and LBBB-T groups

	nQRS (*n* = 24)	LBBB-T (*n* = 24)	*P* value
Demographic details			
Age, years	80.5 ± 6.2	79.6 ± 9.6	0.670
Gender, male	13 (54)	13 (54)	1
Body surface area, m^2^	1.82 ± 0.29	1.86 ± 0.19	0.332
Clinical details			
STS PROM, %	4.5 ± 2.4	5.1 ± 2.8	0.397
STS Morbidity/mortality, %	21.7 ± 7.5	24.5 ± 8.8	0.452
Systolic blood pressure, mmHg	134 ± 25.9	138 ± 18	0.558
Hypertension	12 (57.1)	9 (37.5)	0.383
Cerebrovascular disease	4 (16.7)	4 (16.7)	1
Previous myocardial infarction	5 (20.8)	2 (8.3)	0.220
Chronic obstructive pulmonary disease	6 (25)	5 (20.8)	0.731
Peripheral vascular disease	6 (25)	7 (29.2)	0.745
Diabetes mellitus	4 (16.7)	8 (33.3)	0.182
Any epicardial coronary stenosis >50%	9 (37.5)	13 (54.2)	0.247
Procedural details			
Medtronic CoreValve	17 (71)	17 (71)	1
Pre-implant valvuloplasty	22 (92)	21 (88)	0.637
Post-implant valvuloplasty	6 (25)	6 (25)	1
Femoral access site	20 (83)	23 (96)	0.331
CMR data			
Fibrosis mass, g	3.3 ± 5.7	1.6 ± 3.8	0.081

Data are expressed as mean ± SD or number (%). STS PROM: Society of thoracic surgeons predicted risk of mortality

### Electrocardiographic characteristics

Mean heart rate at baseline was 67 ± 11 and at 6 m was 68 ± 13 bpm. 7 patients (15%) (nQRS *n* = 2, LBBB-T *n* = 5) had atrial fibrillation at baseline. There were no new cases of post-procedural atrial fibrillation. For those in sinus rhythm, mean PR interval remained similar pre and post procedure in both the nQRS group (179 ± 33 to 191 ± 39 ms, *p* = 0.053) and the LBBB-T group (181 ± 30 to 192 ± 37 ms, *p* = 0.171). In the nQRS group there was no change in QRS duration (93 ± 17 to 96 ± 11 ms, *p* = 0.098). In the LBBB-T group, QRS duration increased from 96 ± 14 to 151 ± 12 ms (*p* < 0.001).

### Reverse remodeling according to post-procedure QRS duration

**Fig. 2:**
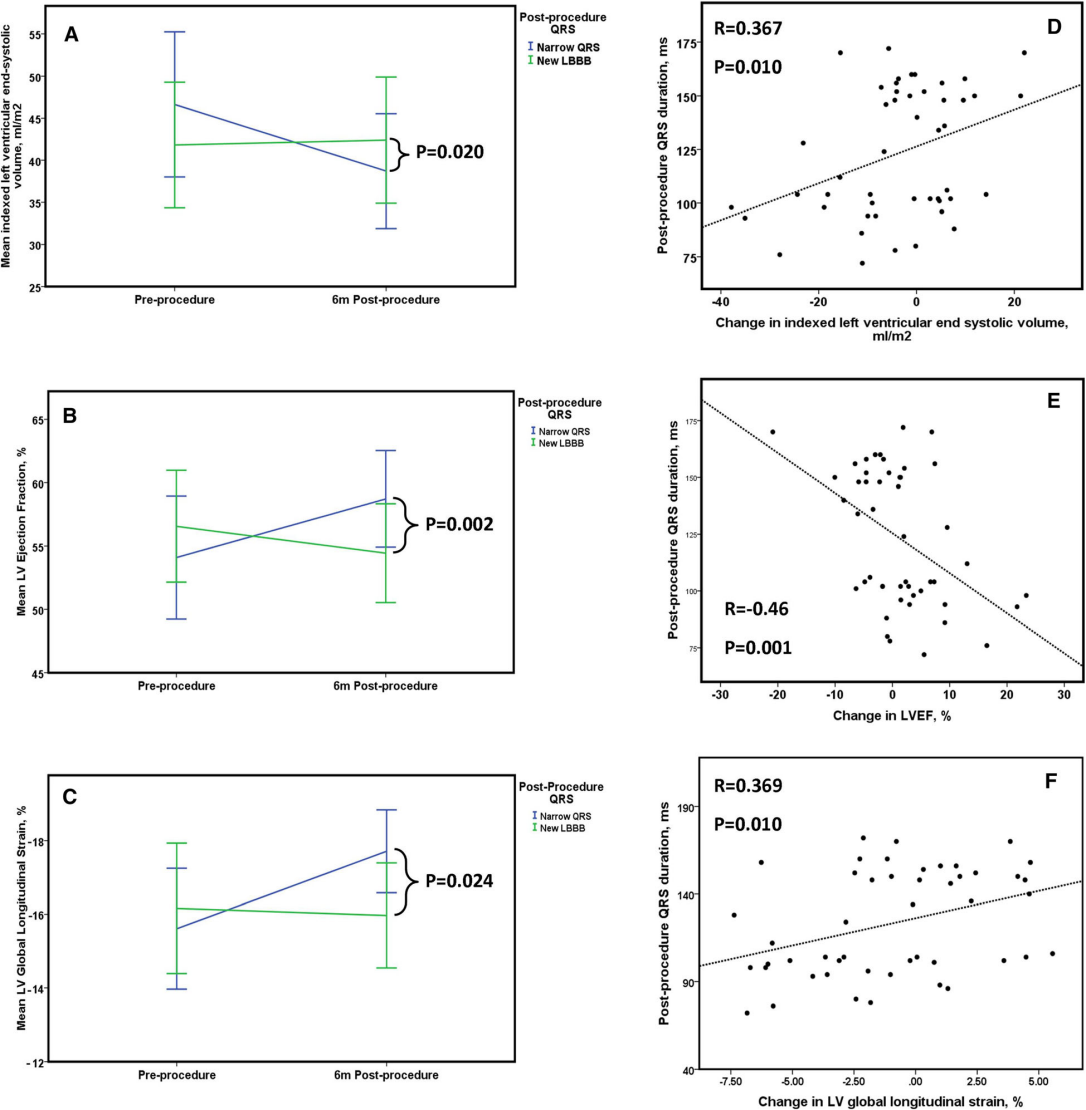
Line graphs depicting change in LVESVi (Panel **a**), LVEF (Panel **b**) and global longitudinal strain (Panel **c**) before and 6 m following TAVR according to post-procedure QRS duration, the vertical lines represent the 95% confidence intervals. Panels **d**, **e** and **f** demonstrate the relationship between post-procedure QRS duration and change in LVESVi, LVEF and GLS

Change in LVEF and GLS was significantly different between the two groups (LVEF: nQRS +4.6 ± 7.8 vs LBBB-T -2.1 ± 6.9%, *p* = 0.002 and GLS: nQRS -2.1 ± 3.6 vs. LBBB-T +0.2 ± 3.2%, *p* = 0.024) (Fig. 2). The change in LVEF was driven by a reduction in indexed LV end systolic volume (LVESVi) in the nQRS group not seen in the LBBB-T group (nQRS -7.9 ± 14.0 vs. LBBB-T -0.6 ± 10.2 ml/m^2^, *p* = 0.02). Pre and post-procedure values for all CMR characteristics can be seen in Table 2. Change in indexed LV mass was similar between the two groups (nQRS -15.9 ± 10.4 vs LBBB-T -13.3 ± 9.6 g/m^2^, *p* = 0.367) as was change in LVEDVi (nQRS -7.3 ± 17.4 vs LBBB-T -3.2 ± 14.5 ml/m^2^, *p* = 0.373). Neither group experienced any change in right ventricular longitudinal function (nQRS 21.7 ± 7.0 to 21.5 ± 6.2 mm, *p* = 0.817, LBBB-T 18.9 ± 5.8 to 18.6 ± 5.8 mm, *p* = 0.773). Post-procedure aortic regurgitant fraction was similar between groups (nQRS 5.4 ± 5.7 vs LBBB-T 5.5 ± 3.3%, *p* = 0.948). There was a significant correlation between change in QRS and change in LVEF (*r* = -0.434, *p* = 0.002) and between change in QRS and change in GLS (*r* = 0.462, *p* = 0.001). The relationship between post-procedure QRS duration and change in LVESVi, LVEF and GLS can be seen in Fig. 2.

**Table 2:** CMR parameters pre and 6 m post-TAVR according to post-procedure QRS status

	nQRS (*n* = 24)	LBBB-T (*n* = 24)	*P* Value
Left ventricular ejection fraction			
Pre-procedure	54.1 ± 11.5	56.6 ± 10.5	0.386
Post-procedure	58.7 ± 9.0	54.4 ± 9.3	0.070
*P* Value	0.010	0.092	
Global longitudinal strain, %			
Pre-procedure	−15.6 ± 3.9	−16.2 ± 4.2	0.638
Post-procedure	−17.7 ± 2.7	−15.9 ± 3.4	0.053
*P* Value	0.009	0.771	
Indexed left ventricular mass, g/m^2^			
Pre-intervention	74.3 ± 14.7	73.3 ± 17.4	0.650
Post-intervention	58.4 ± 12.6	60.0 ± 13.7	0.665
*P* Value	<0.001	<0.001	
Indexed left ventricular end diastolic volume, ml/m^2^			
Pre-intervention	97.8 ± 22.8	93.4 ± 22.1	0.500
Post-intervention	90.5 ± 21.0	90.3 ± 21.0	0.968
*P* Value	0.051	0.298	
Indexed left ventricular end systolic volume, ml/m^2^			
Pre-intervention	46.6 ± 20.4	41.8 ± 17.7	0.458
Post-intervention	38.7 ± 16.2	42.4 ± 17.8	0.523
*P* Value	0.011	0.886	
Indexed left ventricular stroke volume, ml/m^2^			
Pre-intervention	51.2 ± 10.3	51.4 ± 10.5	0.945
Post-intervention	51.8 ± 8.7	47.9 ± 8.5	0.122
*P* Value	0.742	0.035	
Indexed left atrial volume, ml/m^2^			
Pre-intervention	67.9 ± 19.2	72.9 ± 23.3	0.232
Post-intervention	60.0 ± 18.2	67.9 ± 23.8	0.199
*P* Value	0.002	0.180	

*nQRS* narrow QRS post-procedure, *LBBB-T* new LBBB post-procedure

### Inter- and intra-ventricular dyssynchrony

**Fig. 3:**
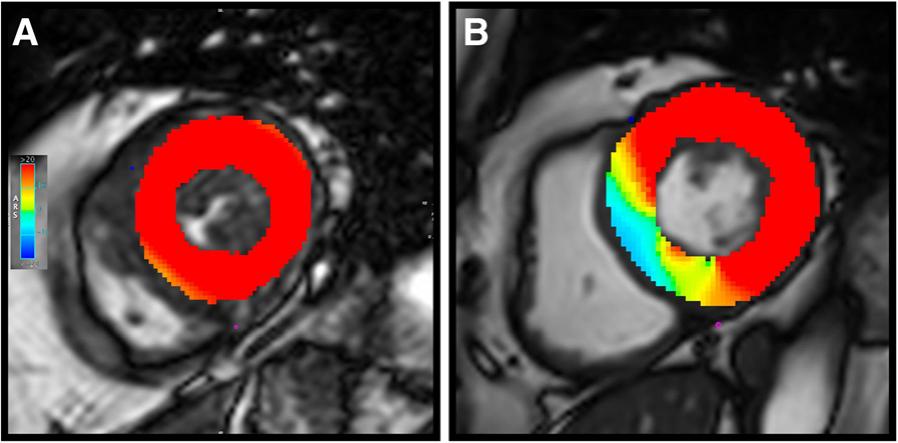
Radial strain in a single mid-ventricular short axis cine. Panel **a** shows the typical contraction pattern in a patient with a nQRS, the red colour depicts positive radial strain occurring in all segments of the left ventricle at end systole. Panel **b** depicts radial strain at end systole in a patient with TAVR-induced LBBB. Peak positive septal radial strain occurs in early systole and therefore by end-systole the septum is relaxing, depicted by the blue colour

As a group as a whole baseline inter- and intra-ventricular dyssynchrony was 68 ± 62 and 54 ± 83 ms respectively. Those that subsequently developed LBBB demonstrated more interventricular dyssynchrony at baseline but had similar baseline intraventricular dyssynchrony (Inter: LBBB-T 88 ± 61 vs. nQRS 48 ± 57 ms, *p* = 0.021, Intra: LBBB-T 74 ± 90 vs. nQRS 35 ± 73 ms, *p* = 0.108). In the nQRS group, TAVR was not associated with an improvement in dyssynchrony (Inter: pre-TAVR 47 ± 57 vs. post-TAVR 23 ± 86 ms, *p* = 0.174, Intra: pre-TAVR 35 ± 73 vs. post-TAVR 7 ± 102 ms, *p* = 0.207). There was evidence of significant inter- and intra-ventricular dyssynchrony in the LBBB-T group at 6 m compared with the nQRS population (Inter: LBBB-T 130 ± 73 vs. nQRS 23 ± 86 ms, *p* < 0.001, intra: LBBB-T 118 ± 103 vs. nQRS 13 ± 13 ms, *p* = 0.001). There was a significant correlation between post-procedure QRS duration and inter- and intra-ventricular dyssynchrony (*r* = 0.57, *p* < 0.001 and *r* = 0.49, *p* = <0.001 respectively). A typical LV contraction pattern in nQRS and LBBB-T can be seen in Fig. 3.

### Predictors of change in LVEF and change in GLS

Baseline variables which may affect cardiac reverse remodeling following TAVR (including clinical, baseline CMR characteristics and post-procedural aortic regurgitation) were analysed to determine univariable predictors of change in LVEF and GLS (Table 3). Baseline LVEF (beta -0.414, *p* = 0.015) and post-procedure QRS (beta -0.422, *p* = <0.001) were independent predictors of change in LVEF at 6 m on multiple regression analysis. Baseline LVEF (beta = -0.502, *p* = 0.001), baseline GLS (beta -1.02, *p* = <0.001) and post-procedure QRS (beta = 0.322, *p* = 0.001) were independent predictors of a change in GLS at 6 m. Infarct pattern LGE at baseline did not impact on post-procedural change in LVEF or change in GLS on univariate analysis.

**Table 3:** Univariate and multiple regression analysis

	Coefficient B	Standard Error	*P* Value	Coefficient B	Standard error	P Value
	Univariate analysis – change in LVEF			Multiple regression analysis – change in LVEF		
Age	−0.201	0.141	0.160			
Sex	2.844	2.246	0.212			
Diabetes mellitus	−1.092	2.624	0.679			
Infarct pattern LGE at baseline	1.647	2.819	0.562			
STS PROM	−0.020	0.448	0.965			
Post-procedure QRS duration	−0.119	0.034	0.001	−0.110	0.028	<0.001
AVAi	7.888	14.638	0.593			
Baseline GLS	−0.963	0.249	<0.001	−0.292	0.319	0.365
Baseline LVEF	−0.393	0.088	<0.001	−0.295	0.117	0.015
Baseline fibrosis mass	−0.007	0.242	0.975			
Post procedure aortic regurgitation fraction	0.089	0.252	0.725			
	Univariate analysis – change in GLS			Multiple regression analysis – change in GLS		
Age	0.090	0.064	0.167			
Sex	−1.161	1.028	0.265			
Diabetes mellitus	−0.467	1.197	0.698			
Infarct pattern LGE at baseline	−0.078	1.291	0.952			
STS PROM	−0.108	0.204	0.600			
Post-procedure QRS duration	0.044	0.016	0.010	0.038	0.011	0.001
AVAi	−4.954	6.658	0.461			
Baseline GLS	−0.588	0.098	<0.001	−0.904	0.122	<0.001
Baseline LVEF	0.094	0.046	0.046	−0.163	0.044	0.001
Post-procedure aortic regurgitation fraction	−0.015	0.116	0.895			
Baseline fibrosis mass	−0.004	0.112	0.970			

*LVEF* left ventricular ejection fraction, *LGE* late gadolinium enhancement, *STS PROM* society of thoracic surgeons predicted risk of mortality, *AVAi* indexed aortic valve area, *GLS* global longitudinal strain

## Discussion

This is the first study using CMR to investigate the impact of TAVR-induced LBBB on cardiac reverse remodeling in a matched population. The main findings of this study are 1) Those with a narrow QRS post-TAVR have better LVEF and GLS compared to those with LBBB-T 6 m post-procedure, 2) Patients with LBBB-T exhibited significant inter- and intra-ventricular dyssynchrony compared with those with narrow QRS and 3) Post-procedure QRS duration remained a significant independent predictor of change in LVEF and GLS following TAVR on multivariable analysis.

### Impact of TAVR-induced LBBB on cardiac reverse remodeling

TAVR-induced LBBB is common, occurring in 16–65% patients depending on valve type [2]. Although predictors of LBBB-T have been extensively studied [2], the impact of LBBB-T on cardiac reverse remodeling is less well described, with studies limited to echocardiographic evaluation and containing a heterogeneous mix of patients. A PARTNER echocardiographic sub-study reported a lower LVEF at 12 months in patients with LBBB on discharge electrocardiogram compared to those with a narrow QRS, however, there was an increased number of those undergoing trans-apical TAVR in the LBBB-T group [6], findings which were replicated in another similar study, again with more undergoing trans-apical TAVR in the LBBB-T group [20]. Tzikas et al. [10] reported an 8% difference in LVEF between those with LBBB-T and nQRS prior to and 6 days following self-expanding TAVR. Longitudinal strain was also non-significantly reduced in those with new conduction abnormalities. Hoffman et al. [11] investigated 90 patients using 2D and speckle tracking trans-thoracic echocardiography prior to and at 1 and 12 months following TAVR. Patients with new conduction defects had a significantly larger LVESVi at 12 months compared with those with a narrow QRS, with less difference in LVEDVi, mirroring the findings in our study. New conduction defects and baseline LVEF were independent predictors of reduction in LVEF at 12 months. The inclusion of patients with trans-apical access in the majority of these studies [6, 11, 20] and those with post-procedural pacemaker insertion [6, 10, 11, 20] is a significant confounder, however, given that trans-apical access has been linked to reduced LVEF in a number of studies [20, 21] and pacing induced LBBB has been shown to cause different patterns of strain to those with idiopathic LBBB [22].

Our study adds further insight into the impact of LBBB-T on cardiac reverse remodeling. The accuracy and reproducibility of CMR means that important differences can be determined using studies 87% smaller than echocardiographically based studies, with only 11 patients per group required to detect a 3% difference in LVEF [13]. Our study groups were matched for clinical and baseline CMR characteristics, all parameters which have been found to strongly influence reverse remodeling following valve intervention [16]. None of the patients in our study received trans-apical TAVR or permanent pacemaker insertion and the unique ability of CMR LGE imaging allowed us to identify and exclude any patients who had a post-procedural myocardial infarction, another factor that may have confounded the earlier echocardiographically based studies. Finally, the two groups experienced similar amounts of post-procedural aortic regurgitation, which is an important modulator of post-TAVR reverse remodeling [23, 24], and which was not reported in most of the echocardiographic studies [6, 10, 11].

### Inter and intra-ventricular dyssynchrony

The novel use of CMR feature tracking allows us to report values for intra- and inter-ventricular dyssynchrony. In LBBB, the normally functioning right bundle conducts the electrical impulse to the right ventricle prompting early right ventricular contraction followed by activation of the interventricular septum and finally lateral wall contraction resulting in inter- and intra-ventricular dyssynchrony. This dyssynchrony leads to the classical abnormal septal motion pattern of contraction seen in LBBB which is felt to impair LV filling and ejection in its own right (Fig. 3). This dysynchronous contraction leads to an increase in LVESVi, as seen in our LBBB-T group and it is this, rather than a change in LVEDVi that is the largest driver of reduction in LVEF. We have also shown that LBBB-T impacted on change in GLS, with no improvement in this group compared to a significant improvement in the nQRS group. Although GLS may be affected by dyssynchrony [25], this, coupled with the reduction in left atrial volume in the nQRS but not the LBBB-T group, and the reduction in LV stroke volume in those with LBBB-T, suggests that the effects of LBBB-T may go beyond that of simple mechanical dyssynchrony.

### Conduction system damage during TAVR

It is well established that TAVR leads to conduction abnormalities [2]. Trauma can occur at multiple time-points during the TAVR procedure; from guidewire manipulation, to during balloon valvuloplasty, device manipulation and valve deployment. It is likely that the different valve designs can cause differing degrees of compression to the conduction system; with the self-expanding Medtronic CoreValve felt to cause more compression of the LV outflow tract than the balloon expandable Edwards Sapien device [26]. The unique design of the mechanically expandable and repositionable Boston Lotus valve with its adaptive seal, may also be associated with more conduction system trauma, although reports to date are limited [27]. Global ischaemia during rapid pacing required for valve deployment may exacerbate the issue [2]. Other procedure-related factors felt to be implicated include pre-implant valvuloplasty, deep implant, low ratio of the annulus:balloon or annulus:prosthesis and operator experience [28].

### Clinical implications

The impact of TAVR-induced LBBB on mortality is a subject of debate, however, it has been shown in many studies to be a predictor of mortality [3–5, 29] and has been associated with increased hospitalisation [6]. Other studies have failed to demonstrate a link [12, 20, 30]. Nonetheless, LVEF is a strong independent predictor of long term survival [31]. Our study has shown that TAVR-induced LBBB is associated with reduced global longitudinal and radial systolic function compared with those with a narrow post-procedure QRS, which could partially explain the link with mortality. Given the adverse effect of TAVR-induced LBBB on cardiac reverse remodeling, restoring inter- and intra-ventricular dyssynchrony using cardiac resynchronisation therapy, could be considered, especially if another conventional indication for device therapy exists. Furthermore, every effort should be made by the operator to reduce the risk of TAVR-induced LBBB given the adverse effects on ventricular remodeling seen. As newer devices are being developed, designs should be focused on minimising damage to the electrical conducting system in order to prevent the deleterious effects on the LV that this entails.

### Study limitations

Although patients were recruited in a prospective manner, they were matched retrospectively and hence the study is prone to the selection bias of this type of study. Patients with LBBB-T were matched according to those factors known to influence cardiac reverse remodeling [16] but other factors may be unaccounted for. Specifically, patients with coronary artery disease and previous myocardial infarction were included in the study, however, numbers in each group were similar and infarct pattern LGE at baseline was not a univariate predictor of change in LVEF or GLS. Group allocation was based on the discharge electrocardiogram and not re-confirmed at 6 months, however, there are evidence to suggest that virtually all those with LBBB at discharge have persistent LBBB at 30 days following self-expanding TAVR [30]. Furthermore, the demonstration of ongoing dyssynchrony at 6 m in the LBBB-T group suggests that the conduction abnormality was persistent. Finally, although adequately powered to detect a difference in reverse remodeling using the accurate technique of CMR, the study is small with a relatively short follow-up period and a larger study with a longer follow-up interval may be helpful to further investigate the impact of TAVR-induced LBBB on cardiac reverse remodeling.

## Conclusion

New LBBB following TAVR is associated with less favourable cardiac reverse remodeling, including effects on LVEF, global longitudinal strain and inter- and intra-ventricular dyssynchrony. In view of this, every effort should be made to minimise the risk of TAVR-induced LBBB especially as TAVR is now being extended to a younger, lower risk population.

## Abbreviations

CMR: Cardiovascular magnetic resonance
GLS: Global longitudinal strain
LBBB: Left-bundle branch block
LBBB-T: TAVR-induced left bundle branch block
LGE: Late gadolinium enhancement
LV: Left ventricular
LVEDVi: Indexed left ventricular end diastolic volume
LVEF: Left ventricular ejection fraction
LVESVi: Indexed left ventricular end systolic volume
nQRS: Narrow QRS
TAVR: Trans-catheter aortic valve replacement
